# Simultaneous Determination of Isothiazolinones and Parabens in Cosmetic Products Using Solid-Phase Extraction and Ultra-High Performance Liquid Chromatography/Diode Array Detector

**DOI:** 10.3390/ph13110412

**Published:** 2020-11-22

**Authors:** Hazim Mohammed Ali, Ibrahim Hotan Alsohaimi, Mohammad Rizwan Khan, Mohammad Azam

**Affiliations:** 1 Department of Chemistry, College of Science, Jouf University, Sakaka 2014, Saudi Arabia; hmali@ju.edu.sa (H.M.A.); ehalshaimi@ju.edu.sa (I.H.A.); 2 Forensic Chemistry Department, Forensic Medicine Authority, Cairo 11441, Egypt; 3 Department of Chemistry, College of Science, King Saud University, Riyadh 11451, Saudi Arabia; mhashim@ksu.edu.sa

**Keywords:** isothiazolinones, parabens, cosmetics, SPE, UHPLC/DAD

## Abstract

Isothiazolinones methylisothiazolinone (MI) and methylchloroisothiazolinone (MCI), and parabens methylparaben (MP), ethylparaben (EP), propylparaben (PP) and butylparaben (BP) are the most common synthetic preservatives. They are all known to be potential skin allergens that lead to contact dermatitis. Thus, the identification of these unsafe chemicals in cosmetic products is of high importance. In the present study, solid-phase extraction (SPE) based on HyperSep reversed-phase C8/benzene sulfonic acid ion exchanger (HyperSep C8/BSAIE) and Sep-Pak C18 sorbents, and ultra-high performance liquid chromatography/diode array detector (UHPLC/DAD) were optimized for the simultaneous determination of MI, MCI, MP, EP, PP and BP in cosmetic products. HyperSep C8/BSAIE and UHPLC/DAD with the eluting solvent mixture (acetonitrile/methanol, 2:1, *v*/*v*) and detection wavelength (255 nm) were found to be the optimal conditions, respectively. The method illustrates the excellent linearity range (0.008–20 μg/mL) with coefficient of determination (R^2^, 0.997–0.999), limits of detection (LOD, 0.001–0.002 μg/mL), precision in terms of relative standard deviation (RSD < 3%, intra-day and <6%, inter-day) when examining a standard mixture at low (0.07 µg/mL), medium (3 µg/mL) and high (15 µg/mL) concentrations. A total of 31 cosmetic samples were studied, achieving concentrations (MI, not detected (nd)-0.89 µg/g), (MCI, nd-0.62 µg/g), (MP, nd-6.53 µg/g), (EP, nd-0.90 µg/g), (PP, nd-9.69 µg/g) and (BP, nd-17.80 µg/g). Recovery values ranged from 92.33 to 101.43% depending on the types of sample. To our knowledge, this is the first specific method which covers the theme and describes background amounts of such preservatives in cosmetics.

## 1. Introduction

Methylisothiazolinone (MI) and methylchloroisothiazolinone (MCI) are the isothiazolinone synthetic biocide which is used as a preservative [1,2,3]. The combination of MI and MCI was used in numerous leave-on and rinse-off formulations comprising skin-care products, bath products, hair products, shampoos, conditioners, facial and eye makeup, face masks, suntan products and wet-wipes products [1,2,3]. MI is currently applied either solely or together with MCI, which comprises a proportion of MCI/MI (3:1). The final product is traded under the name of Kathon, which is sold to the cosmetics manufacturing industries as Kathon CG [4]. Kathon is also applied in the production of papers that usually come into contact with food products. Moreover, this product works as an antimicrobial agent in paper coatings and latex adhesives that interact with food as well [5]. A usual sign of sensitivity to Kathon CG is allergic-contact dermatitis. Sensitization to isothiazolinone groups preservatives was noticed in the 1980s [4,6]. In recent years, the use of isothiazolinone-based preservatives has substantially increased and reported incidence of contact allergy. [6]. In the year 2013, the isothiazolinone-based preservatives were affirmed by the American Contact Dermatitis Society as contact allergen of the year [7]. Following the same year, Cosmetics Europe [8], in coordination with the European Society of Contact Dermatitis [9], suggested to its members that the use of MI in cosmetics, which are intended to stay in long contact with the skin, and cosmetic wet wipes must be ceased. Because of high concerns relating to the potential rising rates of skin sensitivity to MI and MCI, it is highly important to study the presence of such hazardous compounds in cosmetic products available in the markets. 

Parabens are frequently used as antimicrobial and antibacterial preservatives, to prevent the growth of various microbial organisms, particularly fungus and bacteria in cosmetics, drugs and foods [10]. Chemically, parabens are the esters of *p*-hydroxibenzoic acid which contain different alkyl groups, for instance, methylparaben (MP), ethylparaben (EP), propylparaben (PP), butylparaben (BP), benzylparaben (BeP), heptylparaben (HP), isobutylparaben (IBP) and isopropylparaben (IPP) [11]. Among them, MP, EP, PP and BP are the most frequently and repeatedly used in combination with others in the final products [12]. However, the antimicrobial activity increases when using them in a mixture of two or more parabens [12]. Owing to their extensive application, the potential damaging health effects ascribed to parabens could be augmented [13,14]. Although these hazardous compounds have been extensively applied for a long time, a lot of concerns about their effects on human health have remained unsolved. Many researchers have evaluated the influence of severe and persistent exposure of parabens [13,14], as a result parabens effect on the human endocrine system, potential issues in homoeostasis, metabolic syndrome, reproductive systems and breast cancer [13,14,15,16]. Because of their dreadful impact on human health, the World Health Organization and European Commission have established paraben exposure limits in cosmetics and foods [17,18]. The European Union, Health Canada and the United States Food and Drug Administration have recommended the parabens permissible limits of 0.4% (*w*/*w*) and 0.8% (*w*/*w*) in cosmetics [19,20]. Since then, the cosmetics manufacturer has started to produce cosmetics free from MI, MCI and parabens. Nonetheless, adulteration and misbranding of cosmetics takes place by still using the parabens as an ingredient in the cosmetics. Recently, Abad-Gil et al. (2021) reported the presence of isothiazolinone, paraben and alcohol-type preservatives in cosmetic products; they found PE (1800 µg/mL) and MP (590 µg/mL) in facial tonic; MI (1.20 µg/mL), PE (50 µg/mL) and MP (5.20 µg/mL) in shampoo; and PE (1500 µg/mL) and MP (710 µg/mL) in body cream [21]. Alvarez-Rivera et al. (2012) have identified isothiazolinone preservatives in cosmetic products. The products that contained MI and MCI were shampoo (0.38–4.75 and 1.12–9.34 µg/mL), face gel (1.07 and 0.35 µg/mL), hair mask (13.10 µg/mL and <limits of detection (LOD)), dental cream (0.59 µg/mL and <LOD), baby liquid soap (25.8–111 and 0.71–41.8 µg/mL), bath gel (2.05–65.70 and <LOD-3.35 µg/mL), baby shampoo (3.24 and 1.54 µg/mL), makeup (0.83 and 0.18 µg/mL), hair gel (0.72 and 0.22 µg/mL) and baby body milk (1.12–26.10 µg/mL and <LOD) [3]. In other studies, researchers have also reported the presence of isothiazolinone and paraben preservatives in cosmetic products [2,3,21,22,23,24,25,26]. Therefore, the development of sensitive methods to prohibit the adulteration and misbranding of cosmetics is highly needed. To date, there has been no earlier analytical method for the analysis of MI, MCI and parabens in cosmetics or any other matrices. However, many individual methods for MI, MCI, and parabens have been previously reported. The most frequently applied methods for the analysis of MI and MCI were ultra-high performance liquid chromatography–tandem mass spectrometry (UPLC-MS/MS) [2], high performance liquid chromatography-tandem mass spectrometry (HPLC-MS/MS) [3], gas chromatography–tandem mass spectrometry (GC-MS/MS) [27] and high-performance liquid chromatography–ultraviolet (HPLC-UV) [22]. The studied matrices were cosmetics [2,3], shampoo [22], urine [27], milk [28], household products [3], wastewater, surface water, soil, sludge and sediment [29], hygienic consumer products [30], paints [31], food packaging materials [32], cleaning agents and pharmaceuticals [33].

Relating to the determination of parabens, different methods have been reported earlier in various matrices, for instance, HPLC-fluorescence/UV/DAD (cosmetics, toothpaste and mouthwash) [23,24,34], paper spray-MS/MS (cosmetics and drugs) [35], capillary liquid chromatography with UV detection (cosmetics, food and pharmaceuticals) [11], UHPLC-high-resolution mass spectrometry (human urine) [36], UHPLC–MS/MS (human milk) [37] HPLC-MS/MS (domestic sewage) [38] and so on. Some common methods had also been recently reported; Abad-Gil et al., (2021) optimized the simultaneous determination of MI, MCI, 4-hydroxybenzoic acid, phenoxyethanol and MP in cosmetics, using an HPLC/DAD/FL system [21]. In another study, Hefnawy et al., (2017) reported the simultaneous analysis of MI, MP, EP, PP and salicylic acid in cosmetics by monolithic HPLC–PDA [39]. In both methods, the studied compounds and applied methods were found to be different than those used in the current study. The current method (UHPLC/DAD) was found to be rapid, sensitive and economical, especially for the simultaneous determination of MI, MCI, MP, EP, PP and BP in cosmetics. 

Saudi Arabia is the main marketplace for cosmetic products in the Arab and African countries, and it has one of the world’s utmost cosmetics consumption rates. Recently, the Saudi Arabia personal care and beauty market was forecasted to attain $5.5 billion by 2025, rising at a compound annual growth rate of 10.49% during the forecast period (https://www.mordorintelligence.com/industry-reports/saudi-arabia-beauty-and-personal-care-marketm).

Up until now, there has been no earlier analytical system for the identification of MI, MCI and parabens (MP, EP, PP and BP) in cosmetic products by using a single extraction and determination method. Thus, our investigation aimed for the development and validation of a specific method based on solid-phase extraction (SPE) and ultra-high-performance liquid chromatography/diode array detector (UHPLC/DAD) for the simultaneous determination of MI, MCI, MP, EP, PP and BP in cosmetic products. 

## 2. Results and Discussion

### 2.1. Optimization of SPE Method

At present, several extraction methods are available which deal either with MI and MCI [2,3,22] or four potential parabens (MP, EP, PP and BP) [11,23,24,34,35] determination in cosmetics. Thus, the most important aim of the current study was to develop a single extraction and determination method for MI, MCI, MP, EP, PP and BP, which usually co-occur in cosmetic samples. This is the first approach relating to the extraction and determination of these compounds in cosmetics by using a single method. 

Owing to the low amounts of MI, MCI, MP, EP, PP and BP present in the cosmetic samples, the optimization of a reliable method for their analysis is of high importance, and thus, it required a very functional extraction and cleanup system which can eliminate the sample matrix interferences that typically interfere with the determination of target compounds by UHPLC/DAD system. 

**Figure 1:**
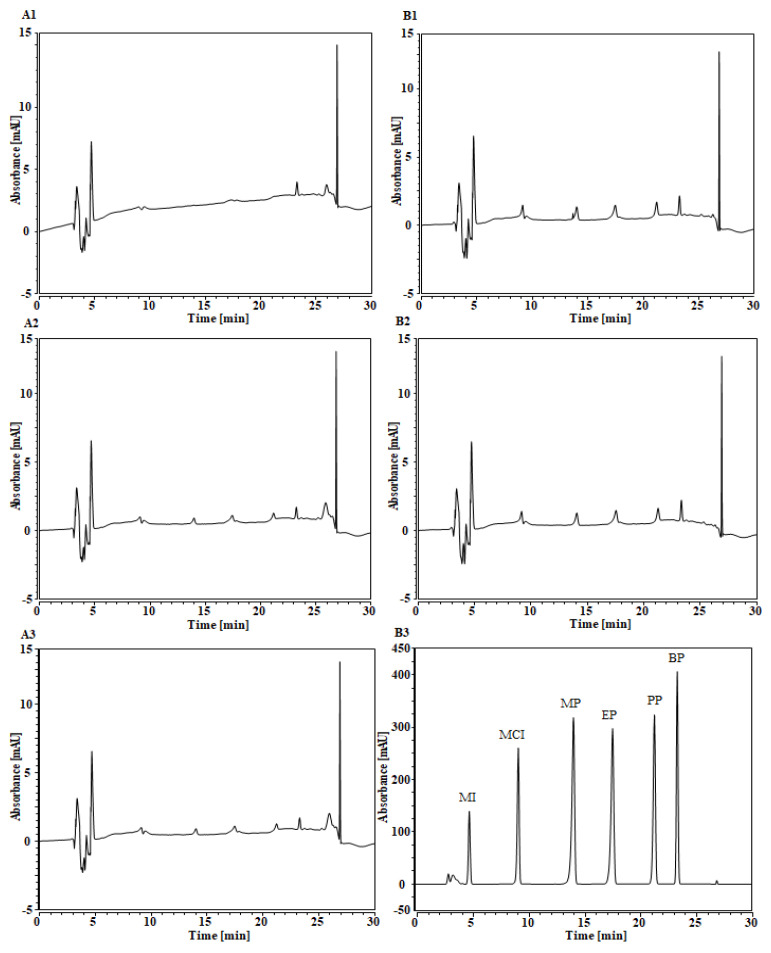
UHPLC/DAD chromatograms of studied compounds: (**A**) Sep-Pak C_18_ and (**B**) HyperSep C_8_/BSAIE. (1) Water/methanol 2:1, *v*/*v*, (2) water/acetonitrile 2:1, *v*/*v* and (3) acetonitrile/methanol 2:1, *v*/*v*.

According to the nature of the analyzed compounds, initially, we selected two types of SPE extraction cartridges, namely HyperSep™ Verify CX Cartridges (Thermo Fisher Scientific, San Jose, CA, USA) of HyperSep C_8_/BSAIE (200 mg/mL) and Sep-Pak C_18_ Classic Cartridge, 360 mg, particle size 55–105 µm (Waters (Milford, MA, USA). Preliminary studies were performed by using a 20 mL mixed solution (3 µg/mL) prepared in methanol and water of all the targeted compounds (MI, MCI, MP, EP, PP and BP). A series of experiments were carried out by the passing sample mixture solution at a controlled flow rate (1 mL/min) through two SPE cartridges separately. Once the sample solution passed completely, the targeted compounds from SPE sorbents were eluted by using different solvent mixtures (10 mL) at various proportions: water/methanol, water/acetonitrile and acetonitrile/methanol. After that, the solution was evaporated under nitrogen gas, until there remained 3 mL of the total solution volume, followed by filtration by using a polytetrafluoroethylene (PTFE) syringe filter (0.45 µm). Finally, the filtrate was injected to UHPLC/DAD, for the determination of MI, MCI, MP, EP, PP and BP. Among them, the SPE cartridge HyperSep C_8_/BSAIE and eluting solvent mixture (acetonitrile/methanol, 2:1, *v*/*v*) were found to be the optimal extraction parameters and used for the analysis of real samples. Figure 1 demonstrates the UHPLC/DAD chromatograms of MI, MCI, MP, EP, PP and BP (standard solution mixture, 3 µg/mL), obtained using different SPE cartridges and eluting solvent mixtures at proportion (2:1, *v*/*v*). It can be observed from Figure 1 that using (A1) water/methanol 2:1, *v*/*v* and Sep-Pak C18; (A2) water/acetonitrile 2:1, *v*/*v* and Sep-Pak C18; and (A3) acetonitrile/methanol 2:1, *v*/*v* and Sep-Pak C18 conditions, the compounds were either not detected or found below LOD. Nevertheless, by using (B1) water/methanol 2:1, *v*/*v* and HyperSep C8/BSAIE; (B2) water/acetonitrile 2:1, *v*/*v* and HyperSep C8/BSAIE; and (B3) acetonitrile/methanol 2:1, *v*/*v* and HyperSep C8/BSAIE conditions, the compounds have been identified in all cases. In B1 and B2 conditions, the compounds were identified with the poor resolution with low peak intensity. However, in B3 conditions, the compounds were identified with excellent resolution and symmetrical with high intensity. 

### 2.2. Optimization of UHPLC/DAD Method

**Figure 2:**
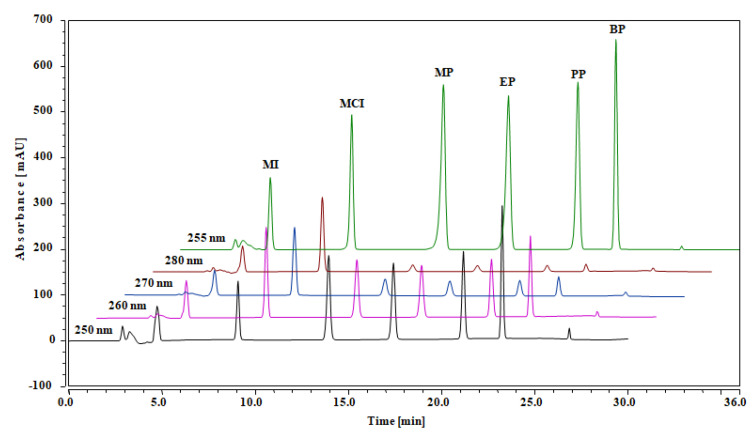
UHPLC/DAD chromatograms of methylisothiazolinone (MI), methylchloroisothiazolinone (MCI), methylparaben (MP), ethylparaben (EP), propylparaben (PP) and butylparaben (BP) obtained at different absorbances, ranging from 250 to 280 nm. The best separation was achieved at absorbance 255 nm.

The most important challenge on the new UHPLC/DAD system was to separate MI, MCI, MP, EP, PP and BP in a single run, with the advantage of high peak resolution, symmetry and short analysis time. Due to differing in their polarity, many determination methods have been reported earlier which deal either with MI and MCI [2,3,22] or parabens (MP, EP, PP and BP) [11,23,24,34,35] in cosmetics. For the optimization of the UHPLC/DAD system, a standard mixture solution (3 µg/mL) was analyzed by using ACCLAIM™ 120 C_8_ analytical column and mobile phase with different solvent proportions, such as water (0.1% formic acid) with acetonitrile/methanol; water (0.05% trifluoroacetic acid) with acetonitrile/methanol; and water (0.1% trifluoroacetic acid) with acetonitrile/methanol. During the assessment of the method parameters, the absorbance was studied in the range of 250–280 nm. The most favorable chromatographic conditions for the analysis of MI, MCI, MP, EP, PP and BP was water (0.1% trifluoroacetic acid) with acetonitrile (mobile phase) and absorbance 255 nm, selected as the final method for real sample analysis. Figure 2 displays the UHPLC/DAD chromatograms obtained at optimal chromatographic conditions. The method offers excellent peak resolution and symmetry and a total analysis time lower than 25 min. The influence of column temperature on analysis was also established in the range from room temperature, 25 °C, to 50 °C, with 5 °C variations. The analysis time was reduced with increasing column temperature beyond 35 °C, which offered a poorer compounds’ separation. Consequently, the column temperature of 35 °C was selected as an optimal condition.

### 2.3. Performance of the Method

The performance of the proposed method was investigated in terms of linearity (R^2^), limit of detection (LOD, signal-to-noise ratio 3:1) and limit of quantification (LOQ, signal-to-noise ratio 10:1), precision (intra- and inter-day) and accuracy. The achieved values have been presented in Table 1 and Table 2. Linearity was determined by analyzing standard mixture at different concentrations, ranging from 0.008 to 20 μg/mL. The analysis was performed in triplicates (*n* = 3). Calibration curves were found to be linear over the broad range of concentrations with the coefficient of determination (R^2^, 0.997–0.999). LOD (signal-to-noise ratio 3:1) and LOQ (signal-to-noise ratio 10:1) values were calculated from the calibration equations using formula 3*standard deviation of the response/slope. LOD and LOQ values were found in the range of 0.001 to 0.002 μg/mL and 0.004 to 0.007 μg/mL, respectively. Precision (intra- and inter-day) was estimated in terms of relative standard deviation (RSD%) and achieved < 3% for intra-day and <6% for inter-day when examining a standard mixture of targeted compounds at concentrations of low (0.07 µg/mL), medium (3 µg/mL) and high (15 µg/mL) levels. Recovery values of targeted compounds were assessed at low, medium and high levels in all of the analyzed samples, and obtained from the added and found concentrations of each compound. The recovery values were achieved between 92.33% and 101.43% depending on the types of sample. The excellent quality conditions were obtained and can be proposed for the determination of these compounds in cosmetics.

### 2.4. Comparison of Proposed Method with the Previous Works

A comparison of the proposed method with the earlier reported analytical methods is presented in Table 3. Earlier methods have individually identified MI and MCI [2] or parabens mixed with other compounds in cosmetics, environmental, biological, pharmaceuticals and personal care samples [11,23,25,33,40], but never the simultaneous identification of the MI, MCI, MP, EP, PP and BP in cosmetics by SPE/UHPLC/DAD. The reason for not identifying these two classes of compounds in a single analysis is the differing polarities, especially when applying a mass spectrometric system. In addition, the reported sample-preparation techniques for the chromatographic determination have only dealt with particular product types. In another approach, Lin et al. (2010) have optimized the UPLC–MS/MS method for the analysis of MI, MCI, 1,2-benzisothiazolinone and 2-octyl-3-isothiazolinone in paper applied for food packaging [41]. The LOD and recovery values were obtained from 0.001 to 0.010 mg/kg and 81.3%, respectively. Fei et al. (2011) studied the MP, EP, PP and BP in cosmetics, by UHPLC/DAD, and obtained LOD (0.12–0.15 mg/mL) and recovery (90.7–97.7%) [26]. Jardim et al. (2015) investigated MP, EP, PP, BP and benzyl paraben in human urine, using UPLC–MS/MS, and found LOD 0.5 ng/mL [42]. These established values [41,42] were also found in good agreement with those archived in the current study. Moreover, on the basis of the achieved outcomes from the current study, the present method could be applied for the determination of MI, MCI, MP, EP, PP and BP in various kinds of matrices. At present, there is no common method available for such kinds of determination, either in sample preparation technique or chromatographic system. The reported SPE/UHPLC/DAD method can simultaneously analyze MI, MCI and parabens (MP, EP, PP and BP) in a single chromatographic method in cosmetic products. The performance of the reported method (linearity, LOD, precision and accuracy) was found to be in good agreement with those reported in earlier works [2,11,23,25,33,40].

### 2.5. Application

**Figure 3:**
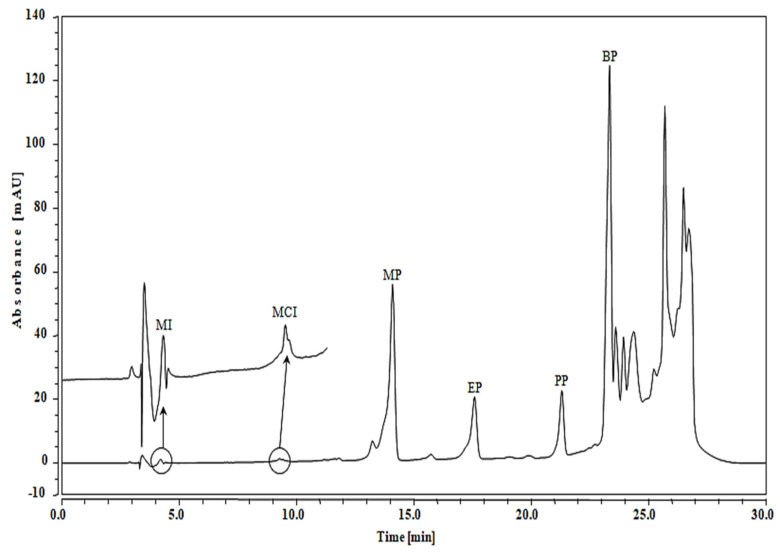
UHPLC/DAD chromatograms identified in perfumed body (dusting) powder (PP_3_, Max) sample.

The practical applicability of the SPE/UHPLC/DAD method was established for the simultaneous determination of MI, MCI, MP, EP, PP and BP in cosmetic products of various trademarks and origin. A total of 31 cosmetic samples (face powder, perfumed body (dusting) powder, wet wipe, shampoo, liquid hand-wash soap and shower gel) were studied (Table 4), achieving the amounts of (MI, nd-0.89 µg/g), (MCI, nd-0.62 µg/g), (MP, nd-6.53 µg/g), (EP, nd-0.90 µg/g), (PP, nd-9.69 µg/g) and (BP, nd-17.80 µg/g) (Table 4). As an example, Figure 3 demonstrates the UHPLC/DAD chromatograms identified in the perfumed body (dusting) powder (PP_3_, Max) sample. Among 31 cosmetic samples, the BP was found in 29 samples, with higher concentrations in shampoo (17.80 µg/g, HS_1_ Pearl touch), followed by MI (27 samples, shampoo HS_3_, SoftCare, 0.89 µg/g), MP (14 samples, face powder FP_7_, Nitrq beauty, 6.53 µg/g), PP (13 samples, perfumed body (dusting) powder PP_1_ Franck Olivier, 9.69 µg/g), MCI (12 samples, face powder FP_2_ kokuryu super summer cake, 0.62 µg/g) and EP (11 samples, perfumed body (dusting) powder PP_3_ Max, 0.90 µg/g). The recovery values ranged from 92.33 to 101.43% depending on the types of sample. The achieved outcomes revealed that the studied cosmetic samples contained these unsafe chemicals in most of the samples even at higher amounts. 

## 3. Materials and Methods

### 3.1. Chemical and Reagents

**Figure 4:**
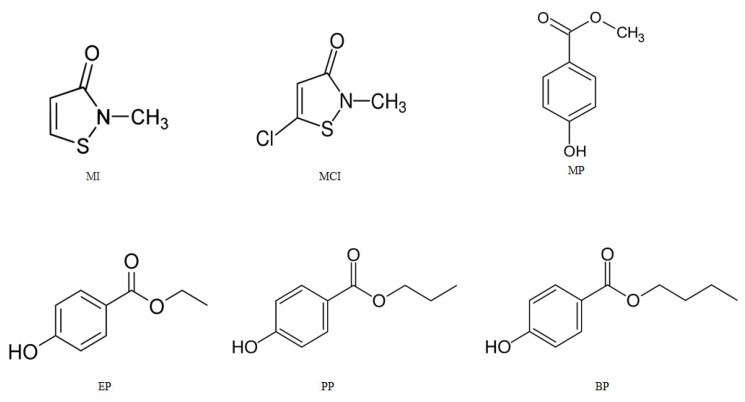
Structures and abbreviation of the studied compounds in cosmetic products.

HPLC grade acetonitrile and methanol were purchased from Sigma-Aldrich (St. Louis, MO, USA). Trifluoroacetic acid, MI, MCI, MP, EP, PP and BP were obtained from Merck (Darmstadt, Germany). All chemicals were of high purity (>99%). The structures of the studied compounds are demonstrated in Figure 4. Ultrapure water was prepared by using a Barnstead^TM^ Smart2Pure^TM^ water purification system (Thermo Scientific, Göteborg, Sweden). Solid-phase extraction cartridges, HyperSep C_8_/BSAIE 200 mg/mL and Sep-Pak C_18_ classic cartridge, 360 mg, particle size 55–105 µm, were purchased from Thermo Fisher Scientific (San Jose, CA, USA) and Waters (Milford, MA, USA), respectively. An ARE Heating Magnetic Stirrer was obtained from VELP Scientifica (Usmate Velate (MB), Italy). Ultrasonic baths, model Bandelin Sonorex Digitec, were obtained from Bandelin electronic (Berlin, Germany). Whatman^®^ qualitative filter paper, Grade 1 circles, diameter 90 mm, was purchased from Merck (Darmstadt, Germany). Polytetrafluoroethylene (PTFE) syringe filter (0.45 μm) was purchased from Macherey-Nagel GmbH (Düren, Germany). 

The individual stock solution was prepared at a concentration of 200 mg/L, by dissolving an adequate weight in methanol, used for further dilutions. To produce the linearity range and calibration curves, standard mixtures of the studied compounds at concentrations ranging from 0.008 to 20 µg/mL were prepared. Standard solutions and cosmetic samples were filtered by a syringe PTFE filter (0.45 μm) (Macherey-Nagel GmbH, Düren, Germany) before being analyzed by the UHPLC/DAD system. 

To assess the SPE efficiency and prevent the matrix influence on peak intensity, retention time and symmetry, the MI, MCI, MP, EP, PP and BP quantification was performed by a standard addition procedure (a quantitative analysis method applied to reduce matrix effects that obstruct with compound measurement signals) consisting of non-fortified (two, zero levels) and fortified (three levels, 50%, 100% and 500%) samples. The levels values demonstrated the increase of compounds in the sample after fortifying. The fortifying of samples was carried out at the start of the extraction method. Cosmetic samples were studied in triplicates (three different extractions of the same sample), and statistical data analysis of the studied samples was performed by means of ANOVA (analysis of variance).

LOD and LOQ were calculated from the calibration equation, i.e., 3*standard deviation of the response/slope. Recovery of MI, MCI, MP, EP, PP and BP was assessed at low, medium and high levels in all of the analyzed samples, and obtained from the added and found concentrations of each analyte. 

### 3.2. Extraction Method 

For the identification and quantification of MI and MCI, and four potential parabens, comprising MP, EP, PP and BP, cosmetics of diverse trademark and country of origin were obtained from cosmetic and pharmacy retail superstore based in Al-Jouf and Riyadh, Saudi Arabia. The sample description was presented in Table 4. Subsequent to purchase, cosmetic samples were immediately stored at 4 °C, and studied at the earliest time, to avoid any chemical loss or contamination. To examine the selective extraction by using SPE method, 0.5 g of cosmetic samples was added to a mixture solution of water and methanol (50:50, *v*/*v*, 20 mL), followed by mixing (10 min) by using a magnetic stirrer. Afterward, the sample mixture was sonicated (10 min) in ultrasonic baths, followed by filtration through Whatman^®^ qualitative filter paper (grade 1 circles, diameter 90 mm). Then, the sample filtrate was eluted through the SPE cartridge (HyperSep C_8_/BSAIE), at a controlled flow rate (1 mL/min). Finally, the analyte was eluted with a mixture solution of acetonitrile and methanol (2:1, *v*/*v*, 10 mL). The sample solution was evaporated, under nitrogen gas, to a final volume of 3 mL. Prior to the analysis by using UHPLC/DAD, the sample extract (3 mL) was filtered through a PTFE syringe filter (0.45 μm). The volume of sample injection was 10 μL. The samples were extracted in triplicates (three different extractions of the same sample). In order to verify the sample contamination and method sensitivity maintained throughout the study, quality control samples were analyzed. Besides this, the sampling steps were carried out with safety measures to reduce sample contamination. 

Because of the complexity of cosmetics preparations, a precise pretreatment of the cosmetic samples is typically needed prior to the identification of these compounds by using the UHPLC/DAD technique. The present SPE method using HyperSep C8/BSAIE cartridge was found to be precise and selective for the analysis of MI, MCI, MP, EP, PP and BP in cosmetics. Nevertheless, in earlier studies, the authors have reported various extraction methods based on ultrasound-assisted extraction, solid-phase microextraction, vortex-assisted dispersive liquid–liquid microextraction and liquid–liquid extraction for the analysis of preservatives in different matrices [3,44,45,46]. These methods were also found to be precise and selective for different types of compounds extracted from different matrices. 

### 3.3. Instrumentation

The sample analysis was performed by using a Dionex UltiMate 3000 UHPLC system (Thermo Scientific, San Jose, CA, USA), comprising a LPG-3400SD binary pump, WPS-3000TSL thermostat autosampler, TCC-3000SD thermostat column compartment and DAD-3000 diode array detector. The data were recorded and analyzed by Chromeleon™ 7.2 Chromatography Data System Software (Thermo Scientific, San Jose, CA, USA).

The chromatographic separation of MI, MCI, MP, EP, PP and BP was achieved through an ACCLAIM™ 120 C_8_ analytical column with the dimensions 150 mm × 2.1 mm and 5 μm of particles size (Thermo Scientific, San Jose, USA). The optimal separation was obtained by using binary mobile phase: water (0.1% trifluoroacetic acid, pH 2.1, solvent A) and acetonitrile (solvent B) at a flow rate of 0.5 mL/min. The gradient mobile phase elution was 0–2 min (B, 12.5%), 2–4 min (B 20–30%), 4–16 min (B, 30–50%), 16–22 min (B, 50–100%), return to its equilibrium conditions and 22–30 min. The column temperature was kept at 35 °C, and the sample injection volume was 10 µL. The column was also washed with a mixture (50:50, *v*/*v*) of methanol and Milli-Q water solution, for five minutes, following the analysis of every ten samples. The optimal detection wavelength was performed in the UV range at 255 nm. 

## 4. Conclusions

A HyperSep C_8_/BSAIE SPE and UHPLC/DAD method for the simultaneous identification of MI, MCI, MP, EP, PP and BP in cosmetic products was optimized and validated, using 31 cosmetic samples of various trademarks and origin. These unsafe chemicals are the most common preservatives that manufacturers frequently apply in such products. In addition, excellent method performance parameters, namely linearity (R^2^, 0.997–0.999), LOD (0.001–0.002 μg/mL), precision (<6%) and accuracy as percent recovery (92.33–101.43%), were achieved. These outcomes revealed that the developed method offers an alternative method for the quality control of MI, MCI, MP, EP, PP and BP in cosmetic products. The present method can be practically used for an extensive range of cosmetic products, for the identification of MI, MCI, MP, EP, PP and BP. For instance, this procedure will be appropriate to screen the frequency of wrong labeling of such unsafe chemicals (MI, MCI, MP, EP, PP and BP) on cosmetics’ ingredients lists. Ingredients labels that are incorrect or omitted can go against clients and health care experts and when seeing for a causative agent to elucidate skin reactions and demanding to evade cosmetics that comprise these potential skin allergens leading to contact dermatitis. 

## Figures and Tables

**Table 1:** Results of linearity (R^2^), limits of detection (LOD) and limits of quantification (LOQ).

Analyte	Linear Range (µg/mL)	R^2^	LOD (µg/g) ± SD	LOQ (µg/g) ± SD
MI	0.005–10	0.997	0.002 ± 0.001	0.007 ± 0.002
MCI	0.005–10	0.998	0.002 ± 0.001	0.007 ± 0.002
MP	0.005–10	0.999	0.001 ± 0.001	0.004 ± 0.001
EP	0.005–10	0.998	0.002 ± 0.001	0.007 ± 0.002
PP	0.005–20	0.997	0.001 ± 0.001	0.004 ± 0.001
BP	0.005–20	0.999	0.001 ± 0.001	0.004 ± 0.001

LOD, signal-to-noise (s/n, 3:1); LOQ, signal-to-noise (s/n, 10:1); SD, standard deviation, obtained from three replicates.

**Table 2:** Accuracy and precision of the proposed (UHPLC/DAD) method.

Analyte	Concentration Added (µg/mL)	Intra-Day			Inter-Day		
		Conc. Found (µg/mL) ± SD	Recovery (%)	RSD (%)	Conc. Found (µg/mL) ± SD	Recovery (%)	RSD (%)
MI	0.07	0.07 ± 0.002	101.14	2.40	0.07 ± 0.004	99.28	5.32
	3	3.01 ± 0.003	100.17	0.11	2.95 ± 0.078	98.37	2.67
	15	14.30 ± 0.035	95.33	0.24	14.30 ± 0.035	95.33	0.24
MCI	0.07	0.07 ± 0.003	98.57	0.67	0.07 ± 0.004	97.14	2.67
	3	2.96 ± 0.003	98.67	0.70	2.95 ± 0.075	98.33	0.78
	15	14.91 ± 0.07	99.40	0.02	14.86 ± 0.02	99.07	0.41
MP	0.07	0.07 ± 0.001	100.14	1.43	0.07 ± 0.003	101.00	4.24
	3	2.99 ± 0.001	99.73	0.34	2.99 ± 0.008	99.53	0.27
	15	14.67 ± 0.003	97.78	0.02	14.66 ± 0.022	97.69	0.15
EP	0.07	0.07 ± 0.001	101.43	1.41	0.07 ± 0.004	98.57	5.80
	3	2.97 ± 0.008	98.90	0.27	2.96 ± 0.001	98.73	0.04
	15	13.9 ± 0.004	92.33	0.03	13.83 ± 0.038	92.20	0.28
PP	0.07	0.07 ± 0.002	100	2.86	0.07 ± 0.003	98.57	4.35
	3	2.97 ± 0.001	99.03	0.04	2.97 ± 0.013	98.90	0.44
	15	14.34 ± 0.001	95.60	0.01	14.32 ± 0.026	95.47	0.18
BP	0.07	0.07 ± 0.001	101.43	1.41	0.07 ± 0.002	100	2.86
	3	3.01 ± 0.003	100.23	0.10	3.01 ± 0.007	100.17	0.23
	15	14.53 ± 0.013	96.87	0.09	14.53 ± 0.007	96.67	0.05

SD, standard deviation; RSD, relative standard deviation.

**Table 3:** Comparison of the proposed method with earlier developed methods.

Sample Type	Analyte	Extraction Method	Determination Method	Analysis Time (min)	Linear Range (ng/mL)	R^2^	LOD (ng/mL)	Precision (RSD%)	R, (%)	Reference
Cosmetics	MI, MCI	Solvent extraction	UHPLC–MS/MS	2.81	0.1–500 (MI), 0.1–1000 (MCI)	0.9997 (MI), 0.9996 (MCI)	43	<7	99–111% (MI), 93–104% (MCI)	[2]
Soil and sediments	MP, EP, PP, BP, IPP, BzP	Ultrasonic-assisted extraction	LC–MS/MS	15	0.6–0.60	0.9993–0.9987	0.04–0.17	<9	83.2–110.2	[40]
Cosmetics, cleaning agents and pharmaceuticals	MI, MCI, BA, SB, MP	Ultrasonic extraction	FLC/UV	27	330–13,330 (MI), 250–10,000 (MCI), 5000–100,000 (BA), 1000–10,000 (SB), 250–10,000 (MP)	0.9996–0.9999	60–4380	0.39–3.45	69–119	[33]
Cosmetics and personal care products	MP, EP, PP, BP, BzP	Fabric-phase sorptive extraction	HPLC/UV	25.27	50–500	0.9955	0.3–0.6	<5	88–122	[23]
Human milk	MP, EP, PP, BP	QuEChERS	HPLC–MS/MS	7.2	0.1–50	0.99	0.04	1–16	83–107	[43]
Food, cosmetics and pharmaceuticals	MP, EP, PP, BP	VA-DLLME-SFO and SA-CPE	CLC/UV	15	100–10,000	0.998	10–30 (VA-DLLME-SFO), 30 (SA-CPE)	<5	-	[11]
Saliva and toothpaste	MP, EP, PP, BP, nBP, iBP	SPE	HPLC/ UV–Vis	15	300–50,000	0.9988–0.9998	100–300	1–6.8	88–113	[25]
Cosmetics	MI, MCI, MP, EP, PP, BP	SPE	UHPLC/DAD	24.7	8–20,000	0.997–0.999	1–2	3–6	92.33–101.43	This work

IPP, isopropyl paraben; BzP, benzyl paraben; BA, benzyl alcohol; SB, sodium benzoate; FLC, fast liquid chromatography; VA-DLLME-SFO, vortex-assisted dispersive liquid–liquid microextraction based on the solidification of a floating organic drop; SA-CPE, salt-assisted cloud point extraction; CLC/UV, capillary liquid chromatography-ultraviolet; -, not described; nBP, n-butyl paraben; iBP, iso-butyl-paraben; R, recovery.

**Table 4:** Amounts of isothiazolinones and parabens obtained in cosmetic products of different brand and origin.

Sample *	Code	Brand	Origin	Concentration (µg/mL ± SD)					
				MI	MCI	MP	EP	PP	BP
Face powder	FP_1_	Max beauty compact powder	China	0.16 ± 0.04	0.23 ± 0.05	0.05 ± 0.02	0.16 ± 0.05	0.41 ± 0.06	0.56 ± 0.06
	FP_2_	Kokuryu super summer cake	China	0.08 ± 0.03	0.62 ± 0.08	0.08 ± 0.02	0.08 ± 0.02	0.33 ± 0.05	nd
	FP_3_	Diamond beauty snake oil	China	0.05 ± 0.01	nd	0.85 ± 0.09	0.13 ± 0.04	3.86 ± 0.15	0.26 ± 0.06
	FP_4_	Kiss beauty compact powder	China	0.07 ± 0.02	nd	nd	nd	1.23 ± 0.10	0.75 ± 0.07
	FP_5_	Bourjois Compact powder	China	0.11 ± 0.03	nd	nd	nd	3.51 ± 0.13	0.92 ± 0.09
	FP_6_	Naked moisturizing and soothing	China	0.13 ± 0.04	nd	nd	nd	0.15 ± 0.04	1.24 ± 0.10
	FP_7_	Nitrq beauty	China	Nd	nd	6.53 ± 0.15	0.18 ± 0.05	1.27 ± 0.11	0.76 ± 0.07
	FP_8_	MaXdona Compact powder	China	0.10 ± 0.03	nd	0.14 ± 0.04	nd	1.76 ± 0.14	0.66 ± 0.06
	FP_9_	Lilianword Compact powder	China	0.08 ± 0.03	nd	0.57 ± 0.07	0.20 ± 0.05	0.55 ± 0.06	0.45 ± 0.05
Perfumed body (Dusting) powder	PP_1_	Franck Olivier	France	0.06 ± 0.01	nd	6.34 ± 0.18	nd	9.69 ± 0.23	1.41 ± 0.13
	PP_2_	Pond’s	India	0.08 ± 0.03	nd	2.02 ± 0.12	0.82 ± 0.10	0.64 ± 0.07	3.03 ± 0.20
	PP_3_	Max	France	0.08 ± 0.02	0.10 ± 0.04	2.16 ± 0.13	0.90 ± 0.10	0.81 ± 0.08	3.73 ± 0.22
Wet wipe	WW_1_	Ribbon	China	0.13 ± 0.05	0.11 ± 0.04	0.05 ± 0.02	0.08 ± 0.02	nd	0.42 ± 0.03
	WW_2_	BabyJoy	UAE	0.10 ± 0.04	0.21 ± 0.06	nd	nd	nd	0.16 ± 0.02
	WW_3_	Good baby	Turkey	nd	nd	nd	0.12 ± 0.03	nd	nd
	WW_4_	Welziadtm	UAE	0.07 ± 0.01	0.31 ± 0.07	0.10 ± 0.03	nd	nd	0.08 ± 0.02
	WW_5_	Dandi	Turkey	0.26 ± 0.02	nd	0.07 ± 0.01	nd	nd	0.25 ± 0.12
	WW_6_	Pafilya	Turkey	0.41 ± 0.03	nd	nd	0.07 ± 0.02	nd	0.16 ± 0.03
	WW_7_	Omay care	Turkey	0.35 ± 0.03	nd	0.16 ± 0.05	nd	nd	0.22 ± 0.04
	WW_8_	Johnson’s	Germany	0.52 ± 0.06	nd	0.54 ± 0.07	0.11 ± 0.03	0.21 ± 0.01	0.11 ± 0.01
	WW_9_	Deema	KSA	0.06 ± 0.01	nd	nd	nd	nd	0.12 ± 0.01
Shampoo	HS_1_	Pearl touch	UAE	0.21 ± 0.04	nd	nd	nd	nd	17.80 ± 1.32
	HS_2_	Perfect cosmetics	UAE	0.27 ± 0.06	nd	nd	nd	nd	13.51 ± 1.20
	HS_3_	SoftCare	China	0.89 ± 0.07	nd	nd	nd	nd	4.94 ± 0.86
Liquid hand wash soap	LS_1_	Soph	Turkey	0.22 ± 0.05	0.11 ± 0.02	nd	nd	nd	5.35 ± 0.92
	LS_2_	Lux	KSA	nd	nd	nd	nd	nd	1.13 ± 0.10
	LS_3_	Gento	KSA	nd	nd	nd	nd	nd	1.15 ± 0.10
	LS_4_	Mada	KSA	0.12 ± 0.04	nd	nd	nd	nd	0.62 ± 0.01
Shower gel	SG_1_	Amalfi	Spain	0.33 ± 0.08	0.31 ± 0.07	nd	nd	nd	0.74 ± 0.06
	SG_2_	Aqua vera	Turkey	0.27 ± 0.06	0.10 ± 0.02	nd	nd	nd	0.11 ± 0.01
	SG_3_	Gian	Turkey	0.11 ± 0.03	0.12 ± 0.02	nd	nd	nd	0.61 ± 0.05

* Samples’ pH values ranged from 5 to 10; SD, standard deviation, calculated from three replicates.
